# CD19 regulates ADAM28‐mediated Notch2 cleavage to control the differentiation of marginal zone precursors to MZ B cells

**DOI:** 10.1111/jcmm.13276

**Published:** 2017-07-14

**Authors:** Yu Zhang, Gaizhi Zhu, He Xiao, Xiaoling Liu, Gencheng Han, Guojiang Chen, Chunmei Hou, Beifen Shen, Yan Li, Ning Ma, Renxi Wang

**Affiliations:** ^1^ Laboratory of Immunology Institute of Basic Medical Sciences Beijing China; ^2^ College of Pharmacy Henan University Kaifeng China; ^3^ Laboratory of Cellular and Molecular Immunology Henan University Kaifeng Henan China; ^4^ Department of Nephrology The 307^th^ Hospital of Chinese People's Liberation Army Beijing China; ^5^ Department of Rheumatology First Hospital of Jilin University Changchun China

**Keywords:** CD19, marginal zone precursors, MZ B cells, Notch2, ADAM28, Foxo1

## Abstract

As the first line of defence, marginal zone (MZ) B cells play principal roles in clearing blood‐borne pathogens during infection and are over‐primed in autoimmune diseases. However, the basic mechanisms underlying MZ B‐cell development are still unclear. We found here that CD19 deficiency blocked the differentiation of marginal zone precursors (MZP) to MZ B cells, whereas CD19 expression in CD19‐deficient MZP rescues MZ B‐cell generation. Furthermore, CD19 regulates Notch2 cleavage by up‐regulating ADAM28 expression in MZP. Finally, we found that CD19 suppressed Foxo1 expression to promote ADAM28 expression in MZP. These results suggest that CD19 controls the differentiation of MZP to MZ B cells by regulating ADAM28‐mediated Notch2 cleavage. Thus, we demonstrated the basic mechanisms underlying the differentiation of MZP to MZ B cells.

## Introduction

Immature B cells leave the bone marrow (BM) and migrate to the spleen, where they finalize their early development by differentiating into mature MZ and follicular (FO) B cells. In the spleen MZ, B cells play principal roles in the first line of defence by clearing blood‐borne pathogens and eliciting a T cell‐independent (TI) immune response 1, 2. They rapidly develop into extrafollicular IgM‐secreting plasma cells within hours of exposure to innate agonists, such as lipopolysaccharide (LPS) and TI antigens 3. On the other hand, a strong reduction in MZ B cells was accompanied by reduced serum IgM and IgG levels and impaired TI immune responses against pathogens 1.

MZ B cells have been found to be involved in various autoimmune diseases. Numbers of MZ B cells increased before any other preclinical signs of disease in lupus‐prone mice 4. In addition, supposedly pathogenic B cells with a predominantly MZ phenotype were found to infiltrate into the thyroid in human Grave's disease or the salivary glands in mice with Sjogren's syndrome 5, 6. However, the mechanisms by which MZ B cells were deregulated require further exploration 7.

Many studies have explored the key aspects of MZ B‐cell generation 8, 9, 10, 11. The B‐cell receptor (BCR) has been reported as important in the development from immature B cells into mature FO or MZ B cells 7. In addition, CD19 lowers the threshold for BcR activation of B cells by acting in concert with CD21. Critically, CD19^−/−^ mice lack MZ B cells suggesting that CD19 plays an important role in MZ B‐cell generation 12. Aside from the BCR and CD19, Notch2 is another key surface receptor required for MZ B‐cell development 13. ADAM‐protease mediated the cleavage of the ectodomain followed by gamma secretase‐mediated release of the Notch intracellular domain (ICN). Analyses of Notch2^−/−^ and DL‐1^−/−^ knockout mice proved that Notch2 signalling is critical for MZ B‐cell development 14, 15. It is however unclear how the CD19, ADAM and Notch2 signals are integrated to determine MZ B‐cell development.

The phenotype of MZ B cell precursors (MZP) has been described as AA4.1^−/low^sIgM^high^CD1d^+^sIgD^high^HSA^+^CD23^+^CD21^high^ 13. However, the differentiation process from MZP to MZ B cells is still unclear. Biochemical characterization of the MZ precursor as compared to MZ B cells should identify signalling pathways crucial for the development of the MZ B subset 16. Thus, in this study we explored the mechanisms underlying the differentiation process from MZP to MZ B cells.

## Materials and methods

### Mice

Seven‐ to nine‐week‐old C57BL/6 (Huafukang Corp., Beijing, China), Foxo1^f/f^ and homozygous CD19^cre^ mice (Nanjing Biomedical Research Institute of Nanjing University, Nanjing, China) were previously described 17 and bred in our animal facilities under specific pathogen‐free conditions. Care, use and treatment of mice in this study were in strict agreement with international guidelines for the care and use of laboratory animals. This study was approved by the Animal Ethics Committee of the Beijing Institute of Basic Medical Sciences.

Lentivirus‐infected cells were adoptively transferred into homozygous CD19^cre^ mice. Mice were randomly placed into the different treatment or no treatment groups without blinding by the investigators.

### Cytometric analysis

All cell experiments were strictly prepared on ice, unless otherwise stated in other specific procedures. Cells (1 × 10^6^ cells/sample) were washed with fluorescence‐activated cell sorting staining buffer (phosphate‐buffered saline, 2% foetal bovine serum or 1% bovine serum albumin, 0.1% sodium azide). All samples were incubated with anti‐Fc receptor Ab (clone 2.4G2; BD Biosciences, San Jose, CA, USA), prior to incubation with other Abs diluted in fluorescence‐activated cell sorting buffer supplemented with 2% anti‐Fc receptor Ab. The samples were filtered immediately before analysis or cell sorting to remove any clumps. Dead cell exclusion/discrimination dyes (Invitrogen, Thermo Fisher Scientific, Waltham, MA, USA) were used to eliminate dead cells from analysis and sorted. For non‐fixed cells, we used DAPI (D1306), SYTOX Blue (S34857), Green (S7020) or Red (S34859); for fixed cells, we use LIVE/DEAD Fixable Blue (L23105), Green (L23101) or Red (L23102). Choice of dead cell exclusion dye depended on the colour combination of fluorochromes within the sample. The following antibodies were purchased from eBioscience, San Diego, CA, USA: fluorescence‐conjugated antimouse B220 (clone no. RA3‐6B2), CD19 (clone no. MB19‐1), CD93 (clone no. AA4.1), CD21 (clone no. 4E3), CD23 (clone no. B3B4), Gr‐1 (RB6‐8C5), CD11b (M1/70), CD43 (eBioR2/60), IgM (eB121‐15F9), IgD (11‐26C) and Notch2 (clone no. 16F11) antibodies. Fluorescence‐conjugated antimouse ADAM28 polyclonal antibody (cat # bs‐5854R) was purchased from Bioss Inc. Cells were acquired using FACSCalibur, FACSAria II, LSRII or LSRFortessa (BD Biosciences) and further analysed using the FlowJo software (Tree Star, Ashland, OR, USA).

### Cell sorting

**Figure 1:**
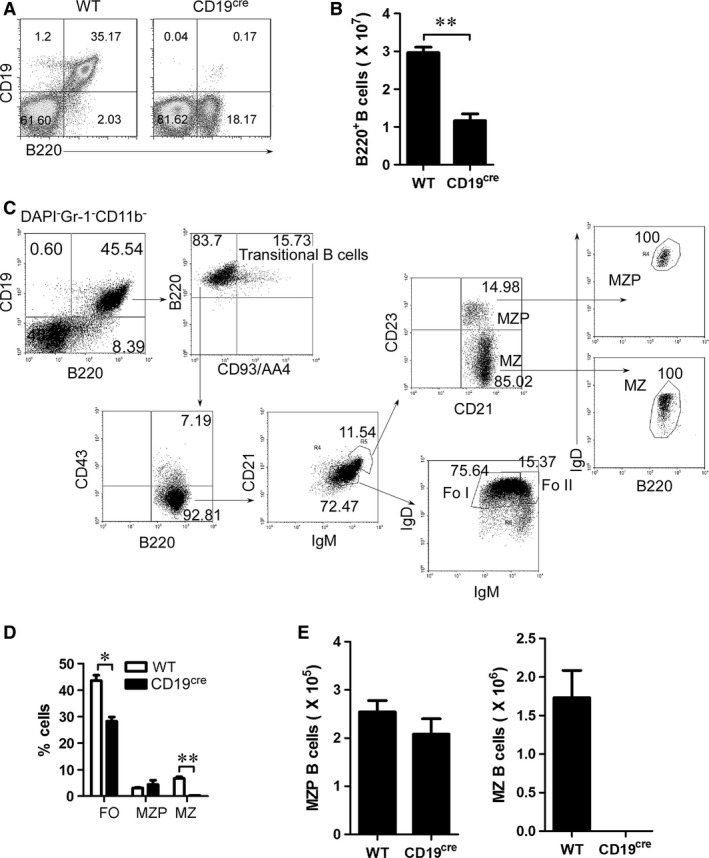
CD19 deficiency blocks marginal zone (MZ) but not MZ precursor (MZP) B‐cell generation. Splenocytes from 7‐ to 9‐week‐old wild‐type (WT) and CD19‐deficient (homozygous CD19^cre^) mice were analysed by flow cytometry (FACS). (**A**) Representative FACS plots indicate percentages of CD19^+^ and B220^+^B cells. (**B**) Absolute number of B220^+^B cells per spleen. (**C**) The gating strategy for analysis and sorting of splenic transitional B cells, marginal zone precursors (MZP), marginal zone (MZ) and follicular (FO) B cells from WT mice. Transitional B cells: DAPI^−^Gr‐1^−^ CD11b^−^CD19^+^B220^+^CD93^+^; MZP B cells: DAPI^−^Gr‐1^−^ CD11b^−^CD19^+^B220^+^CD93^−^ CD43^−^IgM^hi^IgD^hi^ CD21^hi^CD23^+^; MZ B cells: DAPI^−^Gr‐1^−^ CD11b^−^CD19^+^B220^+^CD93^−^ CD43^−^IgM^hi^IgD^lo^CD21^hi^CD23^−^; FO B cells: DAPI^−^Gr‐1^−^CD11b^−^CD19^+^B220^+^CD93^−^ CD43^−^IgM^lo^IgD^hi^CD21^+^CD23^+^. (**D**) The percentage of splenic FO, MZP and MZ B cells from WT and CD19‐deficient (CD19^cre^) mice analysed by FACS based on the analysis strategy described in Figure S1A in total B cells. (**E**) Absolute number of MZP and MZ B cells per spleen. The absolute number of MZ B cells is not presented in CD19‐deficient mice due to the very low number of cells. (**A–E**) Data represent four independent experiments with three individual mice each. Data were analysed by Student's *t*‐test (two tailed) (**B, E**) and Two‐way anova plus Bonferroni post‐tests to compare each column *versus* control column (**D**) and shown as mean ± S.E.M. (*N* = 12 for all groups). *P < 0.05, **P < 0.01.

Splenic B220^+^B were isolated using mouse B220 microbeads (Miltenyi Biotec, Bergisch Gladbach, Germany). The purity of sorted B220^+^B was shown to be >95% by flow cytometric analysis. Multicolour flow cytometry was performed by gating on eGFP^+^ B cells. The gating strategy for analysis and sorting of splenic MZP, MZ, follicular (FO) B cells was employed and slightly revised from the previous strategy 13. Briefly, splenocytes were purificated using lymphocytes separate solution. Splenic lymphocytes were staining with DAPI and antimouse Gr‐1, CD11b, CD19, B220, CD93, CD43, IgM, IgD, CD21 or CD23 antibodies and analysed or sorted by FACS. The surface markers of transitional, FO, MZP, MZ B cells were described in Figure 1C. The purity of sorted cells was shown to be >99% by flow cytometric analysis. All flow cytometry data were acquired with FACSCanto, FACSCantoII or FACSAria (BD Biosciences), gated on live lymphocyte‐sized cells on the basis of forward and side scatter, and analysed using FlowJo software (Tree Star).

### Lentivirus production and Infection

Lentivirus supernatants were prepared by transient cotransfection of 293FT cells (Invitrogen) with mixed package plasmids VSVg, Rev, Gag/Pol and lentiviral constructs pLenti7.3 (V534‐06; Invitrogen) encoding CD19, Notch2IC, ADAM28 or Foxo1 followed by an IRES‐GFP cassette. Viral supernatants were collected after 60–72 hrs. Both Foxo1 shRNA‐ and EGFP‐expressing lentivirus were produced by Shanghai GenePharma., Ltd and described previously 17. For infection, cells were cultured in RPMI1640 supplemented with 10% foetal bovine serum (FBS), 100 U/ml penicillin, 100 μg/ml streptomycin, 2 mM L‐glutamine and 50 μM β‐mercaptoethanol with 1 μg/ml LPS (Sigma‐Aldrich, St. Louis, MO, USA; L2630 from *Escherichia Coli* 0111:B4). Lentiviral supernatants were applied to culture dishes pretreated with RetroNectin (TaKaRa, Kusatsu, Shiga, Japan) and centrifuged at 2,052 g. for 90 min. and then incubated at 37°C in the presence of polybrene (4 μg/ml) for an additional 6 hrs. Cells were then washed and resuspended in fresh media.

### Stimulation of Notch signalling

For stimulated Notch signalling, cells were cultured for 3 days in RPMI1640 supplemented with 10% foetal bovine serum (FBS), 100 U/ml penicillin, 100 μg/ml streptomycin, 2 mM L‐glutamine, and 50 μM β‐mercaptoethanol with 1 μg/ml LPS, and 10 μg/ml of plate‐bound Fc‐Dll1 (R&D Systems, Minneapolis, MN, USA).

### qPCR analysis

All RNA samples were DNA free. cDNA synthesis, RT‐PCR and quantitative PCR (qPCR) analyses were performed as described 18, 19. Each gene‐specific primer pair used for qPCR analysis spanned at least an intron. Primers (Table S1) used for qPCR were purchased from Applied Biosystems Waltham, MA, USA,, and mRNA expression was normalized to the levels of β‐Actin gene.

### Chromatin immunoprecipitation

Chromatin was immunoprecipitated according to the manufacturer's instruction (#9002; Cell Signaling Technology, Danvers, MA, USA) 20, 21, 22. Briefly, sorted cells were crosslinked with 1% (vol/vol) formaldehyde at room temperature for 10 min. and incubated with glycine for 5 min. at room temperature. Cells were then sequentially washed in ice‐cold buffer A and buffer B, followed by digesting with MNase. Nuclear pellet was suspended in ChIP buffer, sheared by sonication with an average size of sheared fragments of about 300 base pairs (bp) to 800 bp. After centrifugation at 9,600 g. for 10 min., sheared chromatin was diluted in ChIP buffer and precleared by addition of protein A/G plus agarose beads (sc‐2003; Sant Cruz Biotech, Santa Cruz, CA, USA) for 1 hr at 4°C. The beads were discarded, and the supernatant was then incubated with one of these antibodies, anti‐Foxo1 or control anti‐IgG (Cell Signaling), at 4°C overnight. At the next day, protein A/G plus agarose beads were added and incubated for 2 hrs at 4°C. Beads were harvested by centrifuge and went through three low salt washes and one high salt wash. Beads were then eluted with ChIP elution buffer. The elutes and input were then added with proteinase K and RNase A and heated at 65°C for 2 hrs to reverse the formaldehyde cross‐link. DNA fragments were purified with column. The relative binding was defined by determining the immunoprecipitation level (ratio of the amount of immunoprecipitated DNA to that of the input sample) and then comparing to corresponding control IgG immunoprecipitation level, which was set as 1.0.

### ADAM28 promoter reporting gene analysis

To clone the murine ADAM28 gene promoter and to construct luciferase reporter gene vectors pGL3 (E1751; Promega, Madison, WI, USA) containing ADAM28 promoter regions, DNA fragments (−2200 ~ +100) of the 5′‐flanking region of murine ADAM28 gene were isolated from genomic DNA of MZP B cells by PCR with ADAM28 promoter primer sets. Empty vector or lentiviral constructs pLenti7.3 encoding Foxo1 followed by an IRES‐GFP cassette and luciferase reporter vector pGL3/ADAM28 promoter were cotransduced into 293T cells. Dual luciferase reporter gene expression was previously described 22, 23 and analysed, and the results were shown as the ratio of firefly to renilla luciferase activity.

### Statistics

Normal distribution of the data was assessed using the Kolmogorov–Smirnov test. Data in all treatment groups were normally distributed. Statistics were analysed using GraphPad Prism (version 5.0; GraphPad Software Inc., La Jolla, CA, USA). The data were shown as mean ± standard error of the mean (S.E.M.). Student's *t*‐test was employed to determine significance between two groups (paired or unpaired), and one‐way or two‐way anova analysis was used to determine significance among several groups. Results were considered statistically significant at *P* < 0.05.

## Results

### CD19 deficiency blocks the generation of MZ but not MZ precursor (MZP) B cells

To explore the mechanisms underlying the differentiation of MZP to MZ B cells, we tested the effect of CD19 deficiency on MZP and MZ B cells. We first determined the effect of CD19 deficiency on total B cells. As expected, CD19 deficiency significantly reduced the total B‐cell percentage and the number in homozygous CD19^cre^ (CD19‐deficient) mice (Fig. 1A and B). To further define the MZ and MZP B cells (Fig. 1C), we used the gating strategy as previously described 13. As expected, we found that CD19‐deficient mice had a profound reduction in both MZ B cells and FO B cells (Fig. 1D, E and Fig. S1A). Interestingly, compared with MZ B cells, MZP B cells were not reduced in CD19‐deficient mice (Fig. 1D, E and Fig. S1A). Together, these results suggest that CD19 deficiency blocks the differentiation process from MZP to MZ B cells.

### MZP B cells express high levels of CD19

**Figure 2:**
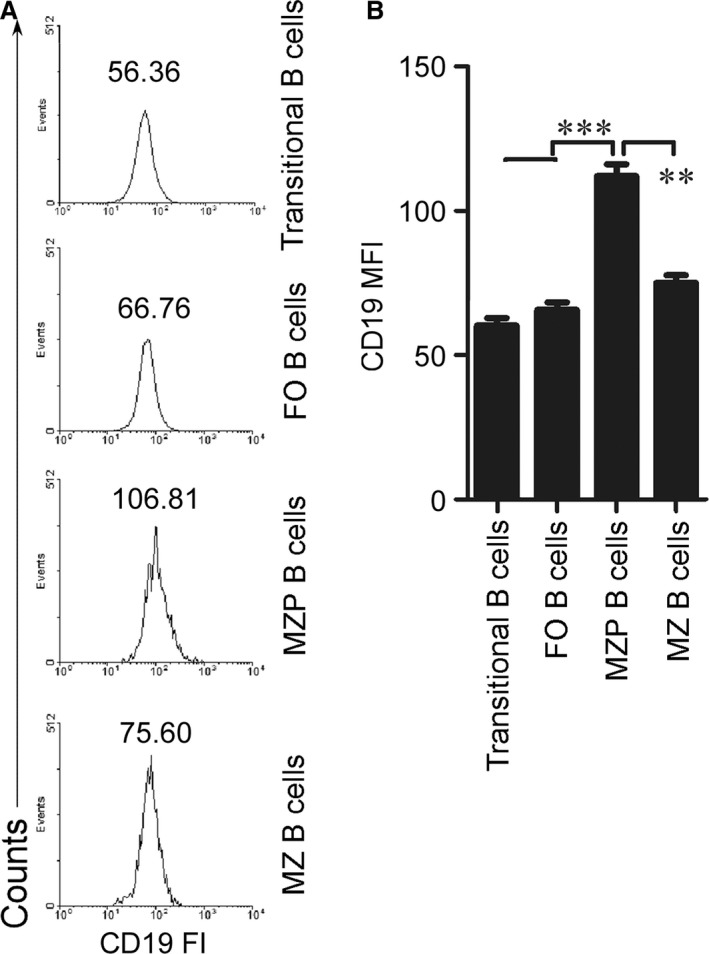
CD19 was highly expressed in MZP B cells. Splenic transitional B cells, MZP, MZ and FO B cells from 7‐ to 9‐week‐old C57BL/6 mice as described in Figure 1C. CD19 fluorescence intensity (FI) on the surface of transitional, FO, MZP and MZ B cells (**A**) was analysed by FACS and CD19 mean fluorescence intensity (MFI) (**B**) is shown. (**A** and **B**) Data represent five independent experiments with three individual mice each. (**B**) Data were analysed by One‐way anova plus Dunnett's multiple comparison test: compare all columns *versus* control column and shown as mean ± S.E.M. (*N* = 15 for all groups). **P < 0.01, ***P < 0.001.

To explore the mechanisms underlying the effect of CD19 on the differentiation process from MZP to MZ B cells, we first determined CD19 expression in MZP and MZ B cells. We used FACS assay and analysed CD19 fluorescence intensity (FI) to determine CD19 expression. The data demonstrated that CD19 FI and mean FI (MFI) on the surface of MZP are the highest among splenic transitional, FO, MZP and MZ B cells (Fig. 2A and B). These results suggest that compared with transitional, FO, MZ B cells, the MZP cells expressed the highest CD19 levels.

### CD19 expression in CD19‐deficient MZP rescues MZ B‐cell generation

**Figure 3:**
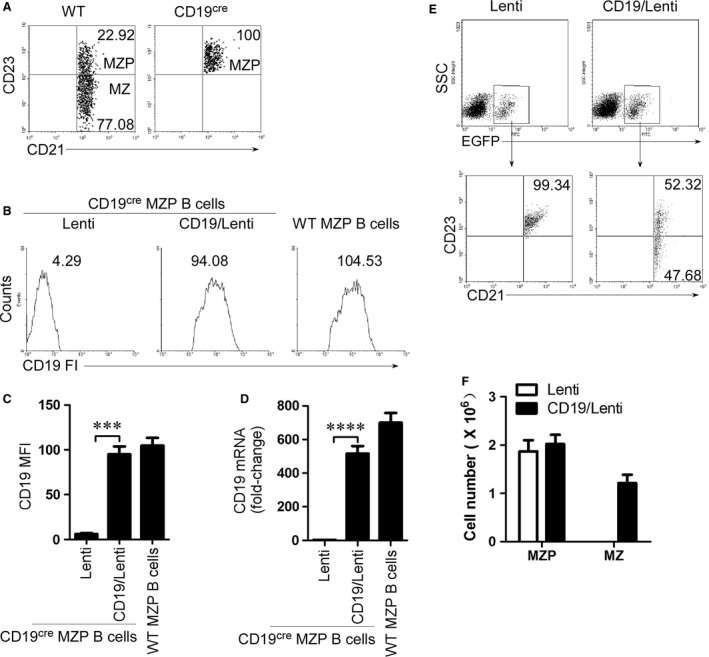
CD19 expression in CD19‐deficient MZP rescues MZ B‐cell generation. (**A**) MZP B cells were sorted from 7‐ to 9‐week‐old CD19‐deficient mice by FACS based on the sorting strategy described in Figure S1B. Sorted MZP B cells from CD19‐deficient mice and MZ/MZP‐containing CD19^+^B220^+^CD93^−^CD43^−^IgM^hi^ CD21^hi^ B cells from WT mice were analysed by CD21 and CD23 staining. (**B–D**) EGFP^+^ cells were sorted by FACS in control lentivirus‐ and CD19‐IRES (internal ribosomal entry site)‐EGFP‐expressing lentivirus‐infected (for 3 days) CD19‐deficient MZP B cells. MZP B cells from WT mice were used as CD19 expression positive control. The expression of CD19 protein and mRNA was identified by FACS (**B** and **C**) and qPCR (**D**). CD19 fluorescence intensity (FI) (**B**), mean FI (MFI) (**C**) and CD19 mRNA level (**D**) are shown. (**E** and **F**) CD19‐IRES‐EGFP‐expressing lentivirus‐infected CD19‐deficient MZP B cells (5 × 10^6^ cells/mouse) were *i.v*. injected into 7‐week‐old CD19‐deficient mice (six mice per group). Spleens were taken from recipient animals on day 7 after injection. Splenic lymphocytes were purified using lymphocyte separation solution and used for EGFP^+^ B‐cell analysis. Gated on EGFP^+^ B cells (upper panel), MZP and MZ B cells (lower panel) were analysed by FACS described in Figure 1C. The percentage (**E**) and absolute numbers per spleen (**F**) of MZP and MZ B cells in EGFP^+^ transferred cells. The absolute number of MZ B cells is not presented in lentivirus‐infected CD19‐deficient MZP B cells due to the very low number of cells. (**A–F**) Data represent three independent experiments with three individual mice each. Data were analysed by one‐way anova (**C** and **D**) and two‐way anova (**F**) plus Bonferroni post‐tests to compare each column *versus* control column and shown as mean ± S.E.M. (*N* = 9 for all groups). ***P < 0.001, ****P < 0.0001.

To test whether CD19 expression in CD19‐deficient MZP rescues MZ B‐cell generation, we first sorted the MZP B cells from CD19‐deficient mice by FACS (Fig. 3A and Fig. S1B). CD19‐deficient MZP B cells were infected with CD19‐IRES (internal ribosomal entry site)‐EGFP‐expressing lentiviruses. The expression of CD19 was confirmed by FACS (Fig. 3B and C) and qPCR (Fig. 3D). The results suggest that CD19 mRNA and protein were expressed in CD19‐deficient MZP B cells. When CD19‐expressing CD19‐deficient MZP B cells were *i.v*. injected into CD19‐deficient mice, the CD19 expression but not control lentivirus infection induced CD19‐deficient MZP (CD21^hi^IgM^hi^CD1d^hi^CD23^+^IgD^hi^) to produce MZ B cells (CD21^hi^IgM^hi^CD1d^hi^CD23^−^IgD^lo^) (Fig. 3E, F and Fig. S2). The data demonstrated that CD19 expression induced MZ B‐cell generation from CD19‐deficient MZP B cells.

### CD19 regulates Notch2 cleavage in MZP B cells

**Figure 4:**
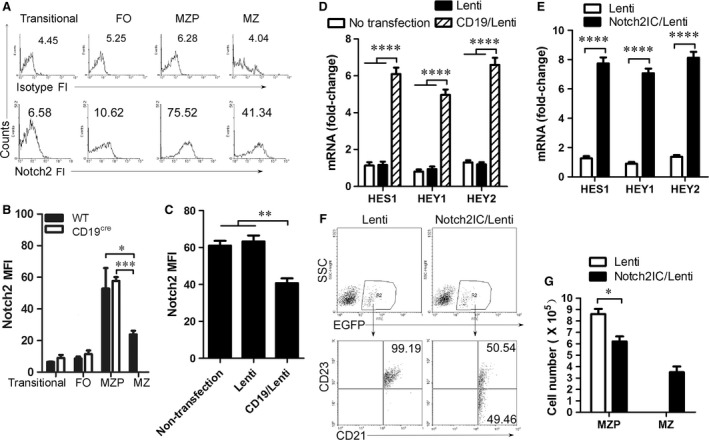
CD19 promotes the differentiation of MZP to MZ B cells by regulating Notch2 cleavage. (**A**) Gated on splenic transitional, FO, MZP and MZ B cells from 7‐ to 9‐week‐old C57BL/6 mice described in Figure 1C, Notch2 fluorescence intensity (FI) was analysed by FACS. FI from isotype antibody staining was used as control. (**B**) Notch2 mean FI (MFI) in splenic transitional, FO, MZP and MZ B cells from 7‐ to 9‐week‐old wild‐type and CD19‐deficient mice was analysed by FACS. A Notch2 MFI histogram is not presented for MZ B cells from CD19‐deficient mice due to the very low number of cells. (**C** and **D**) MZP B cells from 7‐ to 9‐week‐old CD19‐deficient mice were sorted by FACS and infected overnight with control lentivirus‐ and CD19‐IRES‐EGFP‐expressing lentivirus. Non‐ or lentivirus‐infected MZP B cells were cultured for 3 days in the presence of 1 μg/ml LPS and 10 μg/ml of mouse plate‐bound Fc‐Dll1 and analysed by FACS (**C**) and qPCR (**D**), respectively. Notch2 mean fluorescence intensity (MFI) (**C**) and Notch2‐controlled genes including HES1, HEY1 and HEY2 mRNA (**D**) are shown. (**E**) MZP B cells were sorted by FACS from 7‐ to 9‐week‐old CD19‐deficient mice, infected for 3 days with control lentivirus and Notch2IC‐IRES‐EGFP‐expressing lentivirus, and subject to qPCR. Notch2‐controlled genes including HES1, HEY1 and HEY2 mRNA are shown. (**F** and **G**) MZP B cells from 7‐ to 9‐week‐old CD19‐deficient mice were sorted by FACS, infected for 1 day with Notch2IC‐IRES‐EGFP‐expressing lentivirus and (5 × 10^6^ cells/mouse) *i.v*. injected into 7‐week‐old CD19‐deficient mice (six mice per group). Spleens were taken from recipient animals on day 7 after injection. Splenic lymphocytes were purified using lymphocyte separation solution and used for EGFP^+^ B‐cell analysis. Gated on EGFP^+^ B cells (upper panel), MZP and MZ B cells (lower panel) were analysed by FACS described in Figure 1C. The percentage (**F**) and absolute numbers per spleen (**G**) of MZP and MZ B cells in EGFP^+^ transferred cells are shown. An absolute number histogram is not presented for MZ B cells from CD19‐deficient mice due to the very low number of cells. Data represent three independent experiments with six individual mice each. Data were analysed by two‐way anova plus Bonferroni post‐tests to compare each column *versus* control column (**B**,** D**,** E** and **G**) and one‐way anova plus Dunnett's multiple comparison test: compare all columns *versus* control column (**C**) and shown as mean ± S.E.M. (*N* = 18 for all groups). *P < 0.05, **P < 0.01, ***P < 0.001, ****P < 0.0001.

The intracellular form of Notch2 (Notch2IC) drove MZ B‐cell generation in the spleen even in the absence of CD19 24. We tested whether CD19 affected Notch2 expression. We first determined the level of Notch2 expression in splenic transitional, FO, MZP and MZ B cells by FACS. The data demonstrated that Notch2 fluorescence intensity (FI) was higher in MZP than that in transitional, FO and MZ B cells (Fig. 4A). The results suggest that Notch2 is highly expressed in MZP B cells. Compared with WT mice, CD19‐deficient mice had no change in Notch2 expression on the surface of MZP B cells (Fig. 4B). Critically, in Notch ligand Dll1 stimulation condition, CD19 expression in CD19‐deficient MZP B cells infected with CD19‐IRES‐EGFP‐expressing lentivirus reduced Notch2 expression on the cell surface (Fig. 4C). Furthermore, CD19 expression up‐regulated the expression of Notch2‐controlled genes induced by Notch ligand Dll1 (Fig. 4D). In addition, Notch2IC effectively induced the expression of HES1, HEY1 and HEY2 mRNA (Fig. 4E). These results suggest that CD19 promotes the expression of Notch2‐controlled genes by regulating Notch2 cleavage from the surface of MZP B cells to form Notch2IC. To test whether Notch2IC promotes the differentiation from MZP to MZ B cells, Notch2IC‐overexpressing CD19‐deficient MZP B cells were *i.v*. injected into CD19‐deficient mice. Notch2IC overexpression but not control lentivirus infection induced CD19‐deficient MZP (CD21^hi^IgM^hi^CD1d^hi^CD23^+^IgD^hi^) to produce MZ B cells (CD21^hi^IgM^hi^CD1d^hi^CD23^−^IgD^lo^) (Fig. 4F, G and Fig. S3). Thus, Notch2IC effectively induced MZ B‐cell generation from CD19‐deficient MZP B cells. Altogether, these results suggest that CD19 promotes the differentiation of MZP cells to MZ B cells by regulating Notch2 cleavage.

### CD19 mediates Notch2 cleavage by up‐regulating ADAM28 expression

**Figure 5:**
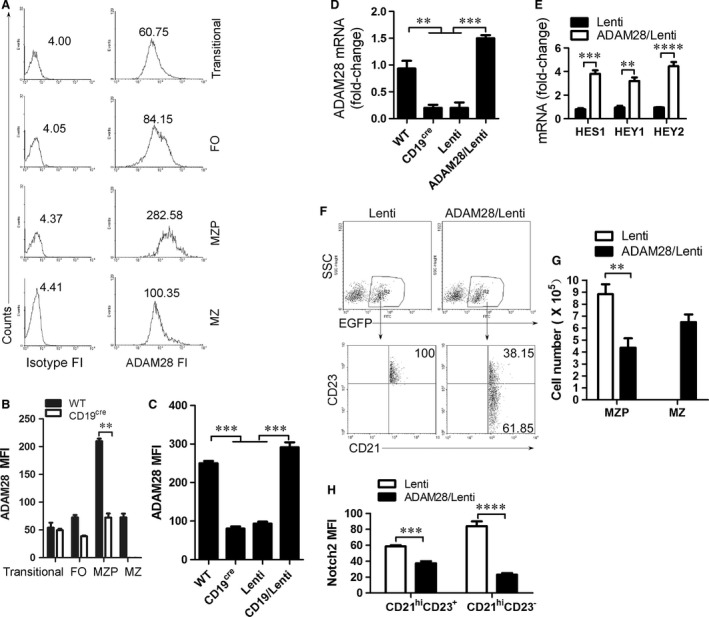
CD19 up‐regulates ADAM28 expression to cleave Notch2. (**A**) Gated on splenic transitional, FO, MZP and MZ B cells from 7‐ to 9‐week‐old C57BL/6 mice described in Figure 1C, ADAM28 fluorescence intensity (FI) was analysed by FACS. FI from isotype antibody staining was used as control. (**B**) ADAM28 mean FI (MFI) in splenic transitional, FO, MZP and MZ B cells from 7‐ to 9‐week‐old WT and CD19‐deficient mice was analysed by FACS. ADAM28 MFI was not presented for MZ B cells from CD19‐deficient mice due to the very low number of cells. (**C** and **D**) ADAM28 MFI (**C**) and mRNA (**D**) in MZP B cells sorted by FACS from 7‐ to 9‐week‐old WT and CD19‐deficient mice, or control lentivirus and CD19‐IRES‐EGFP‐expressing lentivirus‐infected (for 3 days) MZP B cells sorted by FACS from 7‐ to 9‐week‐old CD19‐deficient mice was analysed by FACS (**C**) and qPCR (**D**), respectively. (**E**) Control lentivirus‐ and ADAM28‐IRES‐EGFP‐expressing lentivirus‐infected MZP B cells sorted by FACS from 7‐ to 9‐week‐old CD19‐deficient mice were cultured for 2 days in the presence of 1 μg/ml LPS and 10 μg/ml of mouse plate‐bound Fc‐Dll1, and subject to qPCR. (**F–H**) ADAM28‐IRES‐EGFP‐expressing lentivirus‐infected CD19‐deficient MZP B cells (5 × 10^6^ cells/mouse) were *i.v*. injected into 7‐week‐old CD19‐deficient mice (6 mice per group). Spleens were taken from recipient animals 7 days after injection. Splenic lymphocytes were purified using lymphocyte separation solution and used for EGFP^+^ B‐cell analysis. Gated on EGFP^+^ B cells (upper panel), MZP and MZ B cells (lower panel) were analysed by FACS described in Figure 1c. The percentage (**F**), absolute numbers per spleen (**G**) and (**H**) Notch2 MFI of MZP and MZ B cells in EGFP^+^ transferred cells are shown. Data represent three independent experiments with six individual mice each. Data were analysed by two‐way anova plus Bonferroni post‐tests to compare each column *versus* control column (**B**,** E**, and **G**) and one‐way anova plus Dunnett's multiple comparison test: compare all columns *versus* control column (**C** and **D**) and shown as mean ± S.E.M. (*N* = 18 for all groups). **P < 0.01, ***P < 0.001, ****P < 0.0001.

The ADAM family plays a critical role during Notch2 cleavage to form Notch2IC 25. Previous studies have shown that ADAM10 and ADAM28 may be essential for Notch2‐dependent MZ B‐cell development 26, 27, 28. qRT‐PCR analysis showed that Transitional B cells expressed the high level of ADAM10, whereas MZP B cells expressed the high level of ADAM28 (Fig. S4). Transitional 1 stage (T1) B cells expressing surface ADAM10 were committed to becoming MZB cells *in vivo*, whereas T1 B cells lacking expression of ADAM10 were not 26. To test the role of ADAM28 in the differentiation from MZP to MZ B cells, we examined ADAM28 expression in transitional, FO, MZP and MZ B cells by FACS. ADAM28 fluorescence intensity (FI) analysis showed that ADAM28 is highly expressed in MZP (Fig. 5A). CD19 deficiency reduced ADAM28 on the surface of MZP (Fig. 5B), whereas CD19 expression up‐regulated ADAM28 expression in MZP (Fig. 5C and D). These results suggest that CD19 controls ADAM28 expression in MZP. Furthermore, we showed that ADAM28 overexpression up‐regulated the expression of Notch2‐controlled genes including HES1, HEY1 and HEY2 in CD19‐deficient MZP cells (Fig. 5E). To test whether ADAM28 promotes the differentiation from MZP to MZ B cells, ADAM28‐overexpressing CD19‐deficient MZP B cells were *i.v*. injected into CD19‐deficient mice. ADAM28 overexpression but not control lentivirus infection induced CD19‐deficient MZP (CD21^hi^IgM^hi^CD1d^hi^CD23^+^IgD^hi^) to produce MZ B cells (CD21^hi^IgM^hi^CD1d^hi^CD23^−^IgD^lo^) (Fig. 5F, G and Fig. S5). Finally, we found that Notch2 expression on the surface of ADAM28‐induced MZ B cells was significantly reduced (Fig. 5H). These results suggest that CD19 mediates Notch2 cleavage to induce the differentiation from MZP to MZ B cells by up‐regulating ADAM28 expression.

### CD19 suppresses Foxo1 to promote ADAM28 expression in MZP B cells

**Figure 6:**
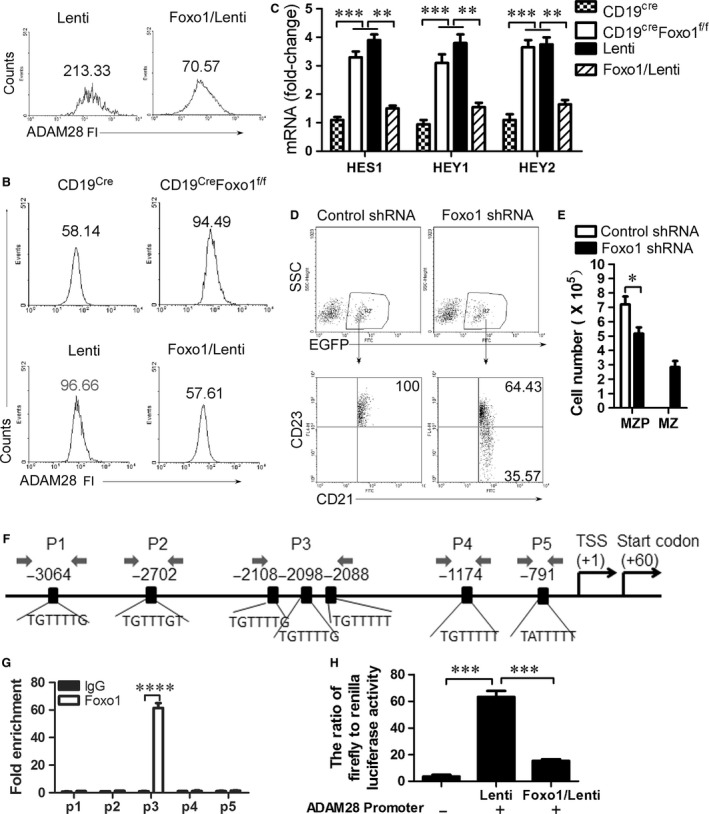
CD19 suppresses Foxo1 to promote ADAM28 expression in MZP B cells. (**A**) MZP B cells from 7‐ to 9‐week‐old WT (C57BL/6) mice were sorted by FACS described in Figures 1C and 3A and infected with Foxo1‐IRES‐EGFP‐expressing lentivirus. On day 3 after infection, ADAM28 fluorescence intensity (FI) was analysed by FACS. (**B**) MZP B cells were sorted by FACS from 7‐ to 9‐week‐old CD19^cre^ and CD19^cre^Foxo1^f/f^ mice. MZP B cells from CD19^cre^Foxo1^f/f^ mice were infected for 3 days with control lentivirus or Foxo1‐IRES‐EGFP‐expressing lentivirus. ADAM28 FI was analysed by FACS. (**C**) MZP B cells were sorted by FACS from 7‐ to 9‐week‐old CD19^cre^ and CD19^cre^Foxo1^f/f^ mice. MZP B cells from CD19^cre^Foxo1^f/f^ mice were infected overnight with control lentivirus or Foxo1‐IRES‐EGFP‐expressing lentivirus. All MZP B cells were cultured for 3 days in the presence of 1 μg/ml LPS and 10 μg/ml of mouse plate‐bound Fc‐Dll1. HES1, HEY1 and HEY2 mRNA was analysed by qPCR. (**D** and **E**) Both Foxo1 shRNA‐ and EGFP‐expressing lentivirus‐infected CD19‐deficient MZP B cells (5 × 10^6^ cells/mouse) were *i.v*. injected into 7‐week‐old CD19‐deficient mice (six mice per group). Spleens were taken from recipient animals on day 7 after injection. Splenic lymphocytes were purified using lymphocyte separation solution and used for EGFP^+^ B cell analysis. Gated on EGFP^+^ B cells (upper panel), MZP and MZ B cells (lower panel) were analysed by FACS described in Figure 1C. The percentage (**D**) and absolute number per spleen (**E**) of MZP and MZ B cells in EGFP^+^ transferred cells are shown. (**F**) Schematic diagram of ADAM28 promoter region illustrating the predicted binding sites and sequences of Foxo1 and the positions of the primer pairs used for ChIP assays. (**G**) ChIP assays of CD19‐deficient MZP B cells using a Foxo1 antibody or control IgG probing for the ADAM28 locus. Quantitative PCR was used to analyse the enrichment, and the fold enrichments are represented from one of three independent experiments. (**H**) Luciferase reporter gene vectors pGL3 containing ADAM28 promoter region (−2200 ~ +100) and Foxo1/lentiviral vector were cotransduced into RAW263.7 cells. Dual luciferase reporter gene expression was analysed, and the results are shown as the ratio of firefly to Renilla luciferase activity. Data represent three independent experiments with six individual mice each (**A–E**) and with six replicates (**G** and **H**). Data were analysed by two‐way anova plus Bonferroni post‐tests to compare each column *versus* control column (**C**,** E**, and **G**) and one‐way anova plus Dunnett's multiple comparison test: compare all columns *versus* control column (**H**) and shown as mean ± S.E.M. (*N* = 18 for all groups) **P < 0.01, ***P < 0.001, ****P < 0.0001.

As we all know, CD19 signalling induces PI3K activation and Foxo1 is a critical effector of the PI3K‐AKT axis in B‐cell development 29. Thus, we tested whether Foxo1 mediated CD19‐controlled ADAM28 expression in MZP. We first showed that Foxo1 overexpression suppressed ADAM28 expression in wild‐type MZP (Fig. 6A and Fig. S6A). In addition, we found that Foxo1 knockout up‐regulated ADAM28 (Fig. 6B, Fig. S6B and C) and Notch2‐controlled genes including HES1, HEY1 and HEY2 (Fig. 6C) in CD19‐deficient MZP, whereas Foxo1 overexpression suppressed ADAM28 expression (Fig. 6B and Fig. S6C) and Notch2‐controlled genes including HES1, HEY1 and HEY2 (Fig. 6C) in both Foxo1‐ and CD19‐deficient MZP. These results suggest that CD19 induced ADAM28‐cleaved Notch2 by suppressing Foxo1 expression. To test whether Foxo1 suppresses differentiation from MZP to MZ B cells, Foxo1‐knocked down CD19‐deficient MZP B cells were *i.v*. injected into CD19‐deficient mice. Foxo1 knocked down but not control lentivirus infection induced CD19‐deficient MZP cells (CD21^hi^IgM^hi^CD1d^hi^CD23^+^IgD^hi^) to produce MZ B cells (CD21^hi^IgM^hi^CD1d^hi^CD23^−^IgD^lo^) (Fig. 6D, E and Fig. S7). These results suggest that CD19 promotes ADAM28‐mediated Notch2 cleavage to induce the differentiation of MZP to MZ B cells by suppressing Foxo1. To further test the possibility that foxo1 may directly suppress the transcription of ADAM28 in MZP, we first examined the association of Foxo1 with the ADAM28 locus by ChIP assays with a panel of primer pairs corresponding to the promoter regions of the ADAM28 locus (Fig. 6F). ChIP analysis showed that Foxo1 was associated with the promoter region of the ADAM28 locus in MZP, suggesting a direct role of Foxo1 in ADAM28 transcription (Fig. 6G). Dual luciferase reporter gene expression analysis demonstrated that Foxo1 directly suppressed ADAM28 expression (Fig. 6H). Altogether, these data suggest that CD19‐mediated Foxo1 controls the differentiation of MZP to MZ B cells by directly regulating ADAM28‐mediated Notch2 cleavage.

## Discussion

Splenic MZ and follicular (FO) B cells are two different populations of mature B cells 30. MZ B cells, located in MZ of the spleen, respond rapidly to T cell‐independent antigen, whereas FO B cells specifically respond to T cell‐dependent antigens 27. As we all know, the synergy between BCR and other signalling pathways plays an important role in both FO and MZ B‐cell development 13. However, these signalling pathways are very complex and poorly understood. We showed here that aside from the BCR, coreceptor CD19 promoted MZ B‐cell generation by up‐regulating a disintegrin and metalloprotease (ADAM28) to cleave Notch2 on the surface of MZ B cells (Figs 4 and 5). The study points to the perfect synergy between BCR‐derived signals and other receptors CD19 and ADAM28, and Notch2 signalling pathways.

The existence of a definable precursor for MZ B cells has been proposed. CD23^+^CD21^high^AA4.1^−/low^sIgM^high^CD1d^+^sIgD^high^HSA^+^ B cells have been described as MZ (CD23^−^CD21^high^AA4.1^−^sIgM^high^CD1d^+^sIgD^low^HSA^+^) B‐cell precursors (MZP) 13. We showed here that CD23^+^CD21^high^IgM^hi^IgD^hi^CD1d^hi^ MZP could differentiate into CD23^−^CD21^high^IgM^hi^IgD^lo^CD1d^hi^ MZ B cells (Figs 3C, 4F, 5F, 6D, Figs S2, S3, S5 and S7).

In recent years, considerable progress has been made in deciphering the function of CD19 in mature FO and MZ B‐cell generation 31. CD19^−/−^ mice exhibit three striking deficiencies in peripheral B‐cell subsets: (*i*) B‐1 cells, (*ii*) MZ B cells and (*iii*) GC B cells 12, 32, 33. We showed here that CD19 deficiency reduced MZ and FO B cells but not MZP (Fig. 1). Further studies demonstrated that CD19 deficiency blocked the differentiation from MZP to MZ B cells (Fig. 3). These data suggest that CD19 is a critical coreceptor for the differentiation of MZP to MZ B cells.

The biochemical basis for signal augmentation by CD19 is becoming clearer, yet still needs more information on the specific and important roles for CD19 as a signal‐transducing component for receptors other than the BCR 31. We showed here that CD19 could regulate Notch2 (Fig. 4), one key cell surface receptor required for MZ B‐cell development 13. The intracellular form of Notch2 (Notch2IC)‐driven MZ B‐cell generation in the spleen was achieved even in the absence of CD19 24. These studies suggest that CD19 mediates a critical signalling pathway in regulating Notch2.

ADAM10 has been shown to be essential for the development of MZ B cells 28. A recent study demonstrated that T1 B cells expressing surface ADAM10 were committed to becoming MZB cells *in vivo*, whereas T1 B cells lacking expression of ADAM10 were not 26. ADAM28 was specifically and significantly expressed only by MZ B cells 27. We showed here that the level of ADAM28 expression was higher than that of ADAM10 in MZ B cells and compared with MZ B cells, MZP expressed higher levels of ADAM28 (Fig. 5A). The specific and significant expression of ADAM28 only by MZP suggests an important role in Notch2‐mediated MZ generation. Our data demonstrated that ADAM28 promoted the differentiation of MZP to MZ by cleaving Notch2 to form Notch2IC. Together, these results suggest that Adam10‐deficiency blocks B‐cell differentiation before the MZP stage, while the CD19‐mediated Adam28 deficiency allows B cells to progress up to the MZP stage. Thus, it is possible that Adam10 may be mandatory for initial commitment towards the MZP stage, while Adam28 would be sufficient/required for further progression beyond this stage.

CD19 signalling induces PI3K activation and Foxo1 is a critical effector of the PI3K‐AKT axis in B‐cell development 29. The PI3K/AKT signalling pathway leads to the inhibition of the Foxo transcription factors 34. Our data are in accordance with these studies suggesting that CD19 suppressed Foxo1 expression (Fig. 6). Further, we demonstrated that Foxo1 directly suppressed ADAM28 expression by binding ADAM28 promoter sites using ChIP analysis and dual luciferase reporter gene expression analysis (Fig. 6F and G). Critically, CD19‐mediated Foxo1 controls the differentiation of MZP to MZ B cells by directly regulating ADAM28‐mediated Notch2 cleavage (Fig. 6). Previous studies suggest that direct activation of mTOR in B lymphocytes confers impairment in B‐cell maturation and loss of MZ B cells 35. Foxo transcription factors have emerged as rheostats that coordinate the activities of Akt and targets of mTOR 34. These studies suggest that Foxo1 may play a critical role in MZ B‐cell development. We demonstrated here that Foxo1 suppressed the differentiation of MZP to MZ B cells by directly suppressing ADAM28 expression (Fig. 6).

We found here that CD19 controls the differentiation process from MZ precursor (MZP) to MZ B cells. Furthermore, CD19 regulates Notch2 cleavage by up‐regulating ADAM28 in MZP. Finally, we found that CD19 suppressed Foxo1 expression to promote ADAM28 expression. In conclusion, CD19 controls differentiation from marginal zone precursor (MZP) to MZ B cells by regulating ADAM28‐mediated Notch2 cleavage. Thus, we demonstrated a basic mechanism underlying the differentiation process from MZP to MZ B cells.

## Conflict of interest

The authors declare no conflict of interest.

## Supporting information


**Figure S1** The gating strategy for analysis and sorting.

**Figure S2** CD19 expression promotes MZ B cell production from CD19‐deficient MZP B cells.

**Figure S3** Notch2IC expression promotes MZ B cell production from CD19‐deficient MZP B cells.

**Figure S4** ADAM10 expresses mainly in transitional B cells, whereas ADAM28 expresses mainly in MZP B cells.

**Figure S5** ADAM28 expression promotes MZ B cell production from CD19‐deficient MZP B cells.

**Figure S6** Foxo1 regulates ADAM28 expression in MZP B cells.

**Figure S7** Lack of Foxo1 promotes MZ B cell production from CD19‐deficient MZP B cells.

**Table S1** Primers for qRT‐PCR.

## References

[1] Pieper K , Grimbacher B , Eibel H . B‐cell biology and development. J Allergy Clin Immunol. 2013; 131: 959–71. 23465663 10.1016/j.jaci.2013.01.046

[2] Martin F , Oliver AM , Kearney JF . Marginal zone and B1 B cells unite in the early response against T‐independent blood‐borne particulate antigens. Immunity. 2001; 14: 617–29. 11371363 10.1016/s1074-7613(01)00129-7

[3] Balazs M , Martin F , Zhou T , *et al* Blood dendritic cells interact with splenic marginal zone B cells to initiate T‐independent immune responses. Immunity. 2002; 17: 341–52. 12354386 10.1016/s1074-7613(02)00389-8

[4] Wither JE , Roy V , Brennan LA . Activated B cells express increased levels of costimulatory molecules in young autoimmune NZB and (NZB × NZW)F(1) mice. Clin Immunol. 2000; 94: 51–63. 10607490 10.1006/clim.1999.4806

[5] Segundo C , Rodriguez C , Garcia‐Poley A , *et al* Thyroid‐infiltrating B lymphocytes in Graves' disease are related to marginal‐zone and memory B‐cell compartments. Thyroid. 2001; 11: 525–30. 11441998 10.1089/105072501750302813

[6] Groom J , Kalled SL , Cutler AH , *et al* Association of BAFF/BLyS overexpression and altered B‐cell differentiation with Sjogren's syndrome. J Clin Invest. 2002; 109: 59–68. 11781351 10.1172/JCI14121 PMC150825

[7] Martin F , Kearney JF . Marginal‐zone B cells. Nat Rev Immunol. 2002; 2: 323–35. 12033738 10.1038/nri799

[8] Bendelac A , Bonneville M , Kearney JF . Autoreactivity by design: innate B and T lymphocytes. Nat Rev Immunol. 2001; 1: 177–86. 11905826 10.1038/35105052

[9] Fagarasan S , Watanabe N , Honjo T . Generation, expansion, migration and activation of mouse B1 cells. Immunol Rev. 2000; 176: 205–15. 11043779 10.1034/j.1600-065x.2000.00604.x

[10] Martin F , Kearney JF . B‐cell subsets and the mature preimmune repertoire. Marginal‐zone and B1 B cells as part of a ‘natural immune memory’. Immunol Rev. 2000; 175: 70–9. 10933592

[11] Martin F , Kearney JF . CD21highIgMhigh splenic B cells enriched in the marginal zone: distinct phenotypes and functions. Curr Top Microbiol Immunol. 1999; 246: 45–50. 10396038 10.1007/978-3-642-60162-0_6

[12] Martin F , Kearney JF . Positive selection from newly formed to marginal zone B cells depends on the rate of clonal production, CD19, and Btk. Immunity. 2000; 12: 39–49. 10661404 10.1016/s1074-7613(00)80157-0

[13] Allman D , Pillai S . Peripheral B cell subsets. Curr Opin Immunol. 2008; 20: 149–57. 18434123 10.1016/j.coi.2008.03.014 PMC2532490

[14] Hozumi K , Negishi N , Suzuki D , *et al* Delta‐like 1 is necessary for the generation of marginal zone B cells but not T cells *in vivo* . Nat Immunol. 2004; 5: 638–44. 15146182 10.1038/ni1075

[15] Saito TS , Chiba M , Ichikawa A , *et al* Notch2 is preferentially expressed in mature B cells and indispensable for marginal zone B lineage development. Immunity. 2003; 18: 675–85. 12753744 10.1016/s1074-7613(03)00111-0

[16] Srivastava B , Lindsley C , Nikbakht N , *et al* Models for peripheral B cell development and homeostasis. Semin Immunol. 2005; 17: 175–82. 15826822 10.1016/j.smim.2005.02.008

[17] Liu X , Zhang Y , Wang Z , *et al* Metabotropic glutamate receptor 3 is involved in B‐cell‐related tumor apoptosis. Int J Oncol. 2016; 49: 1469–78. 27431857 10.3892/ijo.2016.3623

[18] Wang X , Liu X , Zhang Y , *et al* Interleukin (IL)‐39 [IL‐23p19/Epstein–Barr virus‐induced 3 (Ebi3)] induces differentiation/expansion of neutrophils in lupus‐prone mice. Clin Exp Immunol. 2016; 186: 144–56. 27400195 10.1111/cei.12840 PMC5054574

[19] Wang X , Wei Y , Xiao H , *et al* A novel IL‐23p19/Ebi3 (IL‐39) cytokine mediates inflammation in lupus‐like mice. Eur J Immunol. 2016; 46: 1340–50. 27019190 10.1002/eji.201546095 PMC11334612

[20] Jiang XX , Chou Y , Jones L , *et al* Epigenetic regulation of antibody responses by the histone H2A deubiquitinase MYSM1. Sci Rep. 2016; 5: 13755. 10.1038/srep13755 PMC4562257 26348977

[21] Sharabi AB , Aldrich M , Sosic D , *et al* Twist‐2 controls myeloid lineage development and function. PLoS Biol. 2008; 6: e316. 19090621 10.1371/journal.pbio.0060316 PMC2602725

[22] Zhang Y , Wang Z , Xiao H , *et al* Foxd3 suppresses IL‐10 expression in B cells. Immunology. 2017; 150: 478–88. 27995618 10.1111/imm.12701 PMC5343362

[23] Ma N , Xing C , Xiao H , *et al* BAFF suppresses IL‐15 expression in B cells. J Immunol. 2014; 192: 4192–201. 24670802 10.4049/jimmunol.1302132

[24] Hampe F , Ehrenberg S , Hojer C , *et al* CD19‐independent instruction of murine marginal zone B‐cell development by constitutive Notch2 signaling. Blood. 2011; 118: 6321–31. 21795747 10.1182/blood-2010-12-325944

[25] Radtke F , Fasnacht N , MacDonald HR . Notch signaling in the immune system. Immunity. 2010; 32: 14–27. 20152168 10.1016/j.immuni.2010.01.004

[26] Hammad H , Vanderkerken M , Pouliot P , *et al* Transitional B cells commit to marginal zone B cell fate by Taok3‐mediated surface expression of ADAM10. Nat Immunol. 2017; 18: 313–20. 28068307 10.1038/ni.3657

[27] Mabbott NA , Gray D . Identification of co‐expressed gene signatures in mouse B1, marginal zone and B2 B‐cell populations. Immunology. 2013; 141: 79–95. 10.1111/imm.12171 PMC3893852 24032749

[28] Gibb DR , Shikh ME , Kang DJ , *et al* ADAM10 is essential for Notch2‐dependent marginal zone B cell development and CD23 cleavage *in vivo* . J Exp Med. 2010; 207: 623–35. 20156974 10.1084/jem.20091990 PMC2839139

[29] Szydlowski M , Jablonska E , Juszczynski P . Foxo1 transcription factor: a critical effector of the PI3K‐AKT axis in B‐cell development. Int Rev Immunol. 2014; 33: 146–57. 24552152 10.3109/08830185.2014.885022

[30] Martin F , Kearney JF . B1 cells: similarities and differences with other B cell subsets. Curr Opin Immunol. 2001; 13: 195–201. 11228413 10.1016/s0952-7915(00)00204-1

[31] Del Nagro CJ , Otero DC , Anzelon AN , *et al* CD19 function in central and peripheral B‐cell development. Immunol Res. 2005; 31: 119–31. 15778510 10.1385/IR:31:2:119

[32] Rickert RC , Rajewsky K , Roes J . Impairment of T‐cell dependent B‐cell responses and B‐1 cell development in CD19‐deficient mice. Nature. 1995; 376: 352–5. 7543183 10.1038/376352a0

[33] Engel P , Zhou LJ , Ord DC , *et al* Abnormal B lymphocyte development, activation, and differentiation in mice that lack or overexpress the CD19 signal transduction molecule. Immunity. 1995; 3: 39–50. 7542548 10.1016/1074-7613(95)90157-4

[34] Hay N . Interplay between FOXO, TOR, and Akt. Biochim Biophys Acta. 2011; 1813: 1965–70. 21440577 10.1016/j.bbamcr.2011.03.013 PMC3427795

[35] Benhamron S , Tirosh B . Direct activation of mTOR in B lymphocytes confers impairment in B‐cell maturation and loss of MZ B cells. Eur J Immunol. 2011; 41: 2390–6. 21674478 10.1002/eji.201041336

