# Supplementary figures and images for: CD19 regulates ADAM28‐mediated Notch2 cleavage to control the differentiation of marginal zone precursors to MZ B cells
Source: J Cell Mol Med. 2017 Jul 14;21(12):3658–69. doi: 10.1111/jcmm.13276 (PMC5706524; doi:10.1111/jcmm.13276)

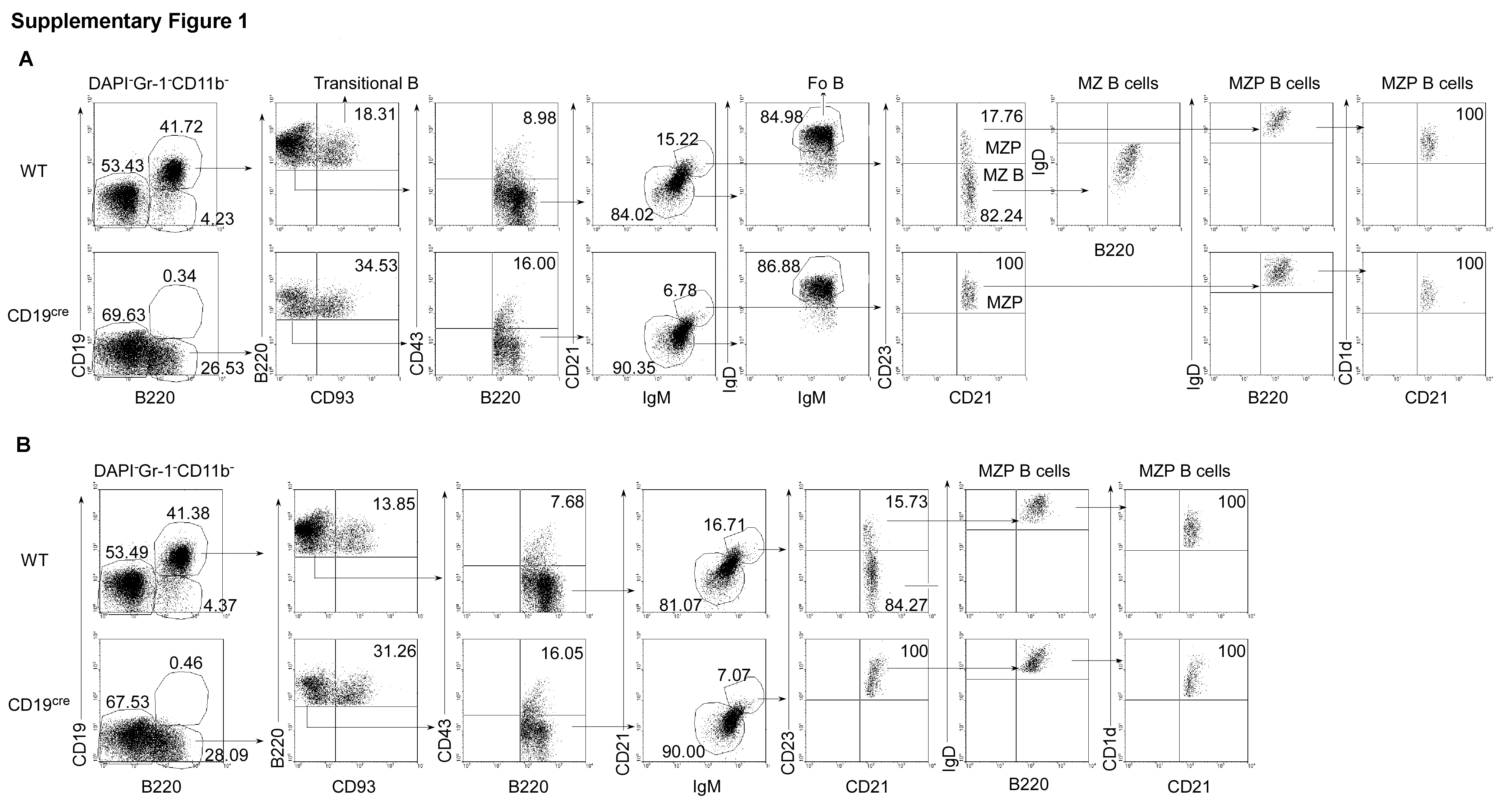

Supplement: Supplementary file 1 — Figure S1 The gating strategy for analysis and sorting. [file JCMM-21-3658-s001.tif]

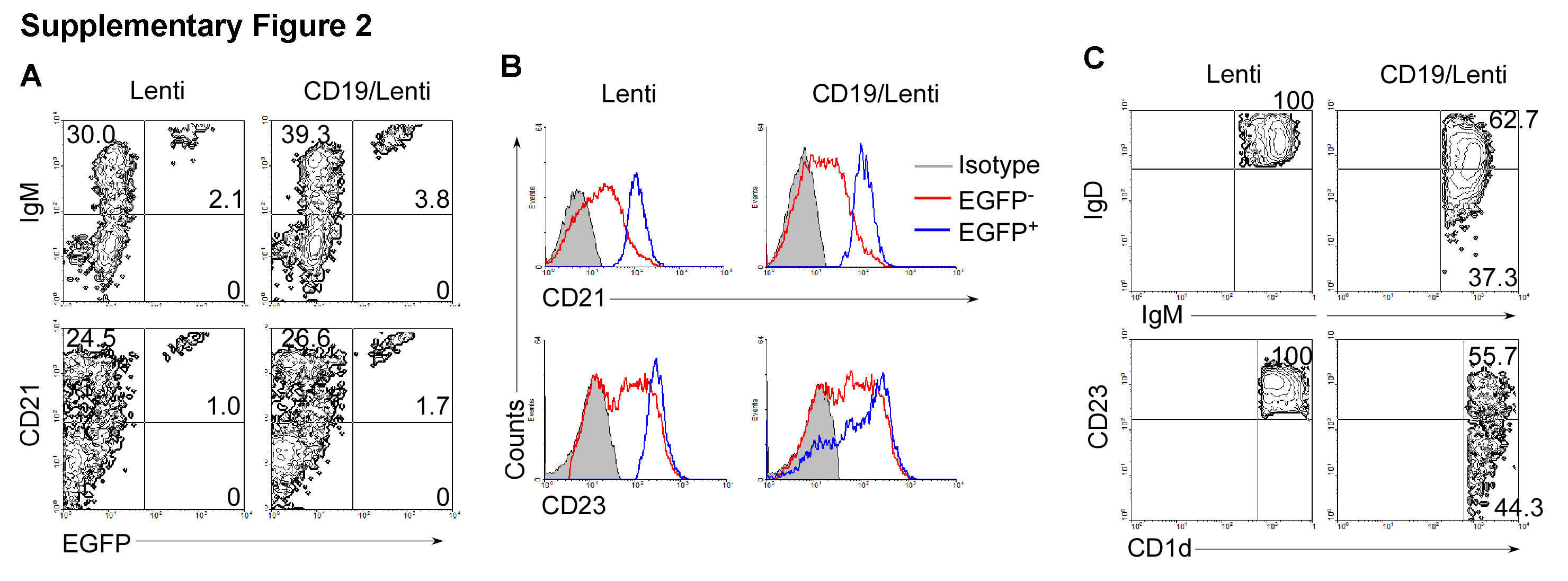

Supplement: Supplementary file 2 — Figure S2 CD19 expression promotes MZ B cell production from CD19‐deficient MZP B cells. [file JCMM-21-3658-s002.tif]

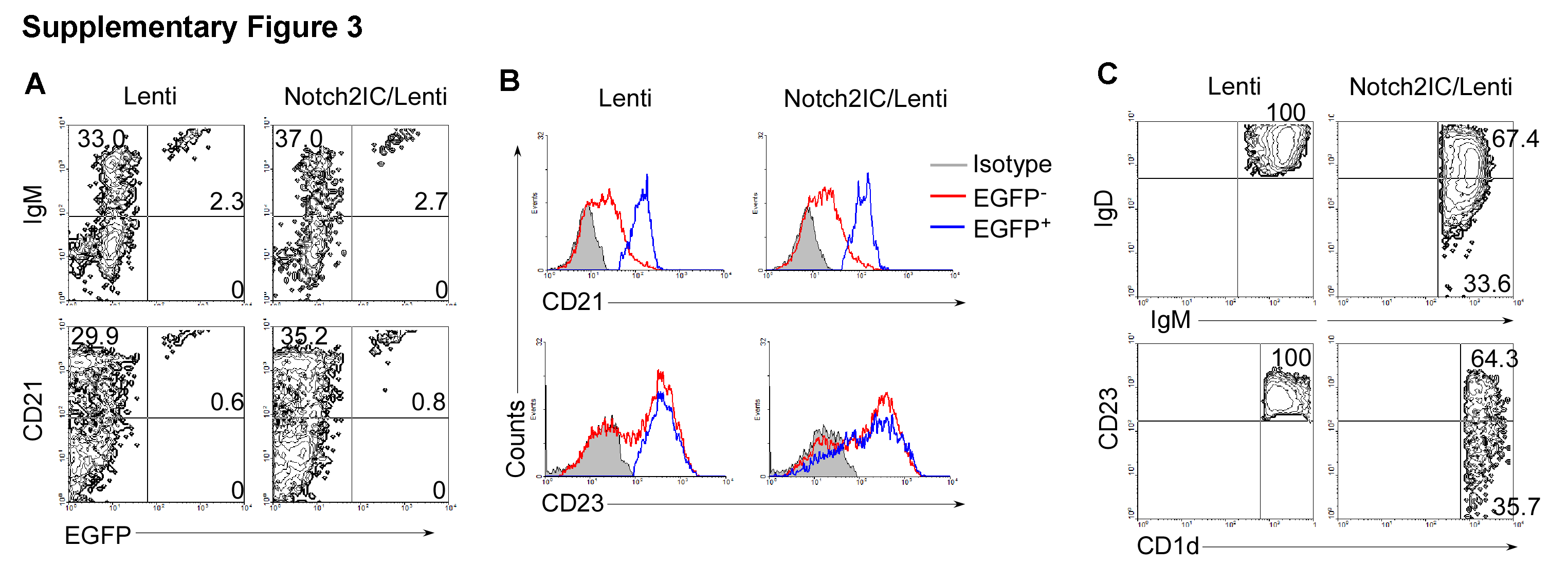

Supplement: Supplementary file 3 — Figure S3 Notch2IC expression promotes MZ B cell production from CD19‐deficient MZP B cells. [file JCMM-21-3658-s003.tif]

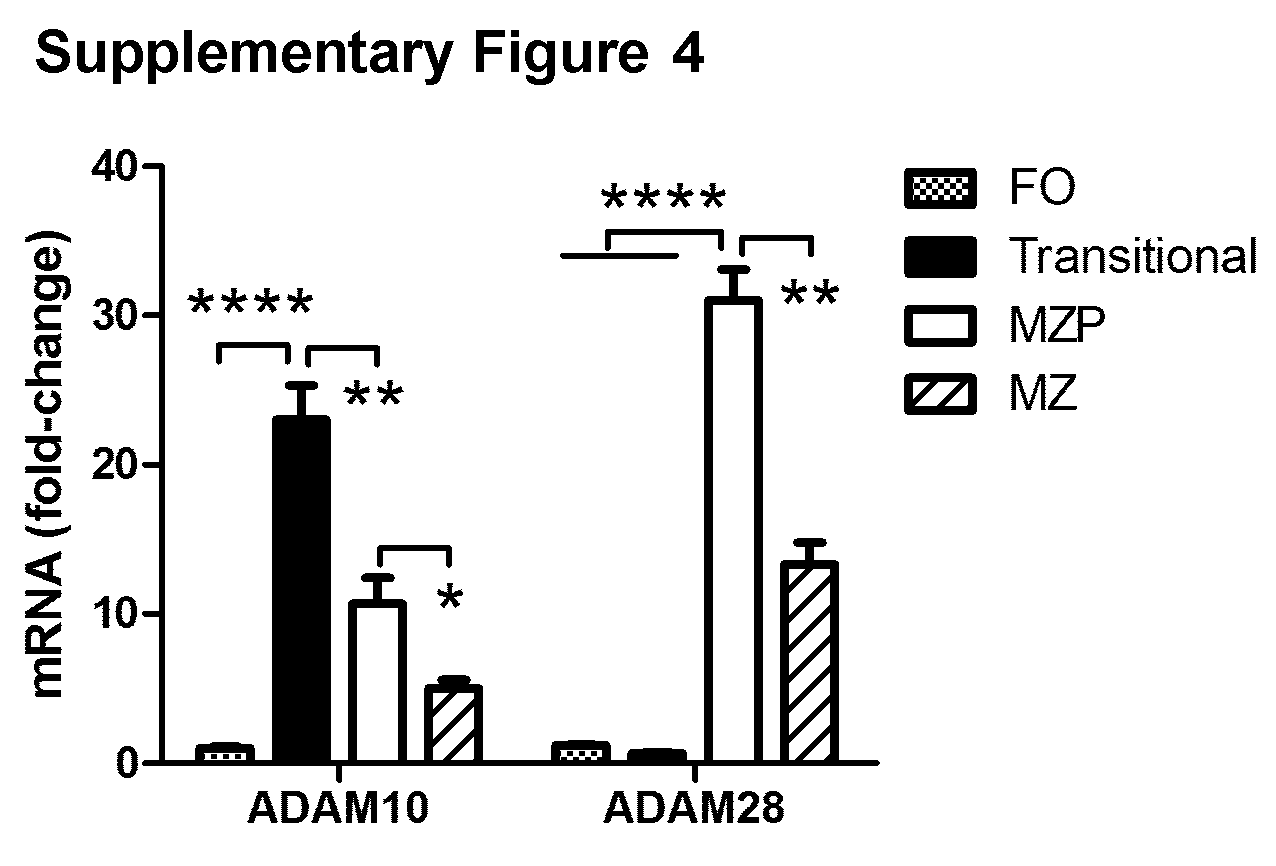

Supplement: Supplementary file 4 — Figure S4 ADAM10 expresses mainly in transitional B cells, whereas ADAM28 expresses mainly in MZP B cells. [file JCMM-21-3658-s004.tif]

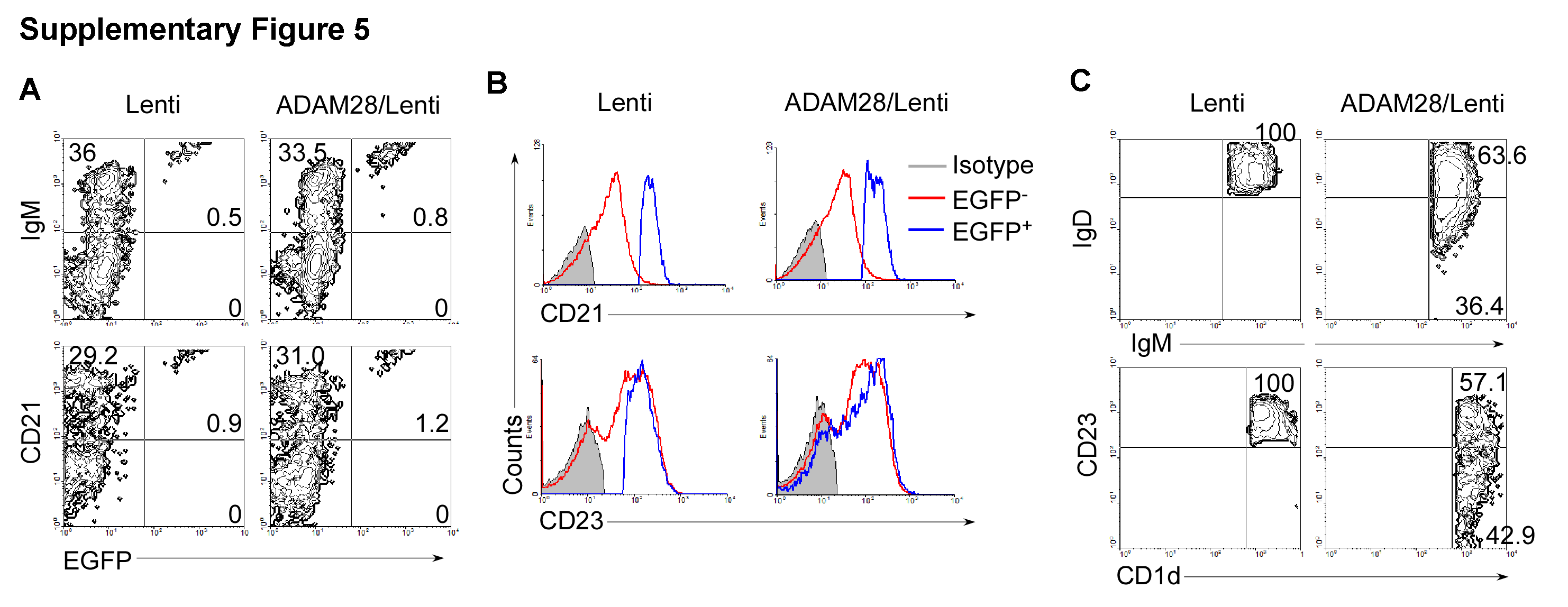

Supplement: Supplementary file 5 — Figure S5 ADAM28 expression promotes MZ B cell production from CD19‐deficient MZP B cells. [file JCMM-21-3658-s005.tif]

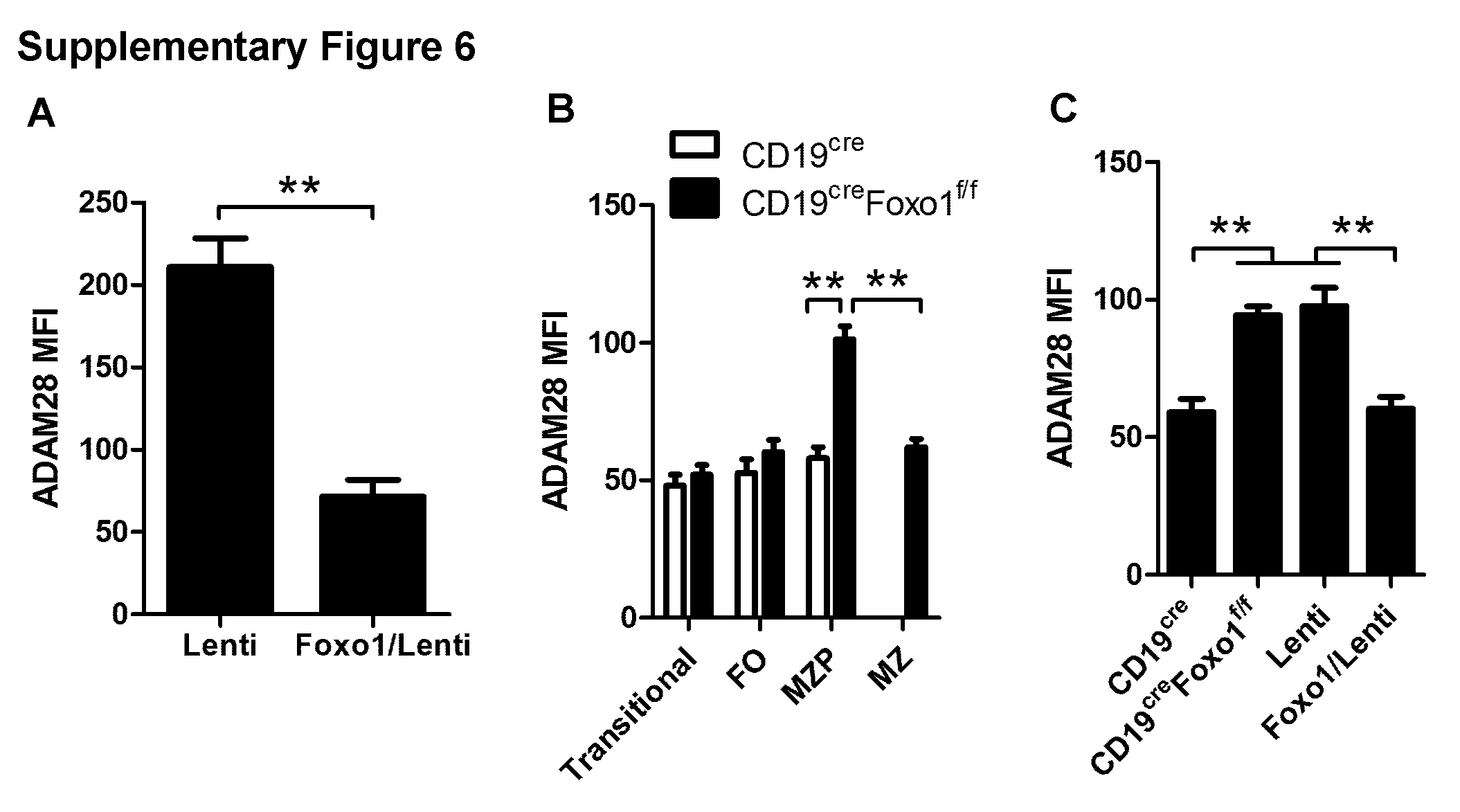

Supplement: Supplementary file 6 — Figure S6 Foxo1 regulates ADAM28 expression in MZP B cells. [file JCMM-21-3658-s006.tif]

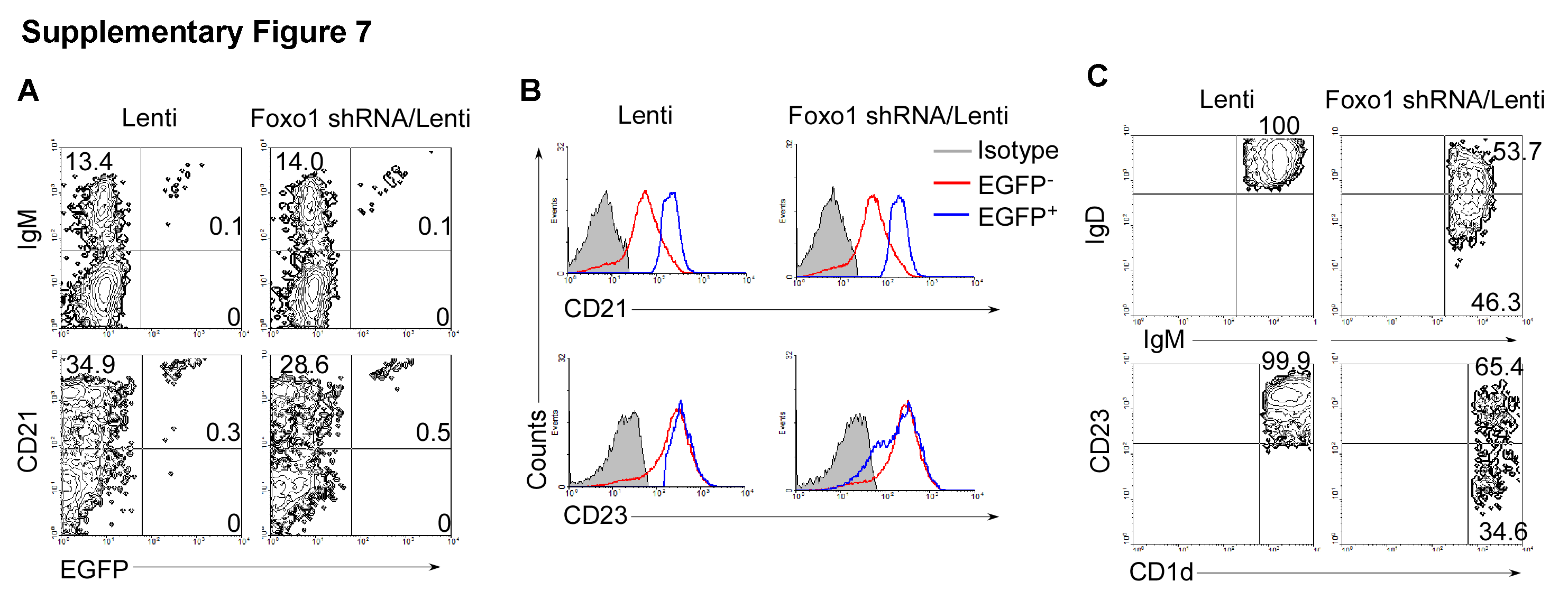

Supplement: Supplementary file 7 — Figure S7 Lack of Foxo1 promotes MZ B cell production from CD19‐deficient MZP B cells. [file JCMM-21-3658-s007.tif]
