# Supplementary material for: CD19 regulates ADAM28‐mediated Notch2 cleavage to control the differentiation of marginal zone precursors to MZ B cells
Source: J Cell Mol Med. 2017 Jul 14;21(12):3658–69. doi: 10.1111/jcmm.13276 (PMC5706524; doi:10.1111/jcmm.13276)
Supplement: Supplementary file 8 — Table S1 Primers for qRT‐PCR. [file JCMM-21-3658-s008.docx]

**Table S1.** Primers for qRT-PCR.

| Mus musculus gene name | Forward and reverse primer sequence |
| --- | --- |
| CD19 | Fwd: 5’-GCCACAGCTTTAGATGAAGGCAC-3’  Rev: 5’-CATCCACCAGTTCTCAACAGCC-3’ |
| Hes1 | Fwd: 5’-GGAAATGACTGTGAAGCACCTCC-3’  Rev: 5’-GAAGCGGGTCACCTCGTTCATG-3’ |
| Hey1 | Fwd: 5’-CCAACGACATCGTCCCAGGTTT-3’  Rev: 5’-CTGCTTCTCAAAGGCACTGGGT-3’ |
| Hey2 | Fwd: 5’-TGAAGATGCTCCAGGCTACAGG-3’  Rev: 5’-CCTTCCACTGAGCTTAGGTACC-3’ |
| ADAM10 | Fwd: 5’-GAAGATGGTGTTGCCGACAG-3’  Rev: 5’-TTTCCATACTGACCTCCCAGC-3’ |
| ADAM28 | Fwd: 5’-TCAGCACCTGTCAAGGACTACG-3’  Rev: 5’-CACCACAGTTGCTCTTCTCCTG-3’ |
