# Supplementary material for: CD19 regulates ADAM28‐mediated Notch2 cleavage to control the differentiation of marginal zone precursors to MZ B cells
Source: J Cell Mol Med. 2017 Jul 14;21(12):3658–69. doi: 10.1111/jcmm.13276 (PMC5706524; doi:10.1111/jcmm.13276)
Supplement: Supplementary file 9 [file JCMM-21-3658-s009.docx]

**Supplementary Figure Legends**

**Fig. S1** The gating strategy for analysis and sorting. (**A**) The gating strategy for analysis of splenic transitional B cells, marginal zone precursors (MZP), marginal zone (MZ), and follicular (FO) B cells from WT mice and CD19-deficient (CD19^cre^) mice. (**B**) The gating strategy for sorting of splenic MZP from CD19-deficient (CD19^cre^) mice. WT mice were used as the control.

**Fig. S2** CD19 expression promotes MZ B cell production from CD19-deficient MZP B cells. CD19-IRES-EGFP-expressing lentivirus-infected CD19-deficient MZP B cells (5 × 10^6^ cells/mouse) were i.v. injected into 7-week-old CD19-deficient mice (6 mice per group). Spleens were taken from recipient animals on days 7 after injection. (**A**) The percentage of IgM^hi^EGFP^+^ and CD21^hi^EGFP^+^ cells in total splenic lymphocytes, CD21 and CD23 expression between EGFP^+^ and EGFP^-^ B cells (**B**) and the percentage of IgM^hi^IgD^hi^, IgM^hi^IgD^lo^, CD1d^hi^CD23^+^, and CD1d^hi^CD23^-^ cells in EGFP^+^ B cells (**C**) were analyzed by FACS. (**A-C**) Data represent three independent experiments with three individual mice each.

**Fig. S3** Notch2IC expression promotes MZ B cell production from CD19-deficient MZP B cells. MZP B cells from 7-9-week-old CD19-deficient mice were sorted by FACS, infected for 1 day with Notch2IC-IRES-EGFP-expressing lentivirus and (5 × 10^6^ cells/mouse) i.v. injected into 7-week-old CD19-deficient mice (6 mice per group). Spleens were taken from recipient animals on days 7 after injection. (**A**) The percentage of IgM^hi^EGFP^+^ and CD21^hi^EGFP^+^ cells in total splenic lymphocytes, CD21 and CD23 expression between EGFP^+^ and EGFP^-^ B cells (**B**) and the percentage of IgM^hi^IgD^hi^, IgM^hi^IgD^lo^, CD1d^hi^CD23^+^, and CD1d^hi^CD23^-^ cells in EGFP^+^ B cells (**C**) were analyzed by FACS. (**A-C**) Data represent three independent experiments with three individual mice each.

**Fig. S4** ADAM10 expresses mainly in transitional B cells, whereas ADAM28 expresses mainly in MZP B cells. MZP B cells from 7-9-week-old WT (C57BL/6) mice were sorted by FACS described in Figures 1C and 3A, and subject to qRT-PCR. Relative mRNA levels are normalized by β-Actin mRNA expression and calculated relative to ADAM10 mRNA expression seen in FoB cells, set as 1. Data were analyzed by Two-Way ANOVA plus Bonferroni post-tests to compare each column vs control column (N=10 for all groups), *P < 0.05, **P < 0.01, ****P < 0.0001.

**Fig. S5** ADAM28 expression promotes MZ B cell production from CD19-deficient MZP B cells. ADAM28-IRES-EGFP-expressing lentivirus-infected CD19-deficient MZP B cells (5 × 10^6^ cells/mouse) were i.v. injected into 7-week-old CD19-deficient mice (6 mice per group). Spleens were taken from recipient animals 7 days after injection. (**A**) The percentage of IgM^hi^EGFP^+^ and CD21^hi^EGFP^+^ cells in total splenic lymphocytes, CD21 and CD23 expression between EGFP^+^ and EGFP^-^ B cells (**B**) and the percentage of IgM^hi^IgD^hi^, IgM^hi^IgD^lo^, CD1d^hi^CD23^+^, and CD1d^hi^CD23^-^ cells in EGFP^+^ B cells (**C**) were analyzed by FACS. (**A-C**) Data represent three independent experiments with three individual mice each.

**Fig. S6** Foxo1 regulates ADAM28 expression in MZP B cells. (**A**) MZP B cells from 7-9-week-old WT (C57BL/6) mice were sorted by FACS described in Figures 1C and 3A, and infected with Foxo1-IRES-EGFP-expressing lentivirus. On day 3 after infection, ADAM28 expression was analyzed by FACS and ADAM28 mean fluorescence intensity (MFI) was shown. (**B**) ADAM28 expression on the surface of transitional, FO, MZP and MZ B cells from 7-9-week-old CD19^cre^ and CD19^cre^Foxo1^f/f^ mice was analyzed by FACS, and ADAM28 mean fluorescence intensity (MFI) was shown. (**C**) MZP B cells were sorted from 7-9-week-old CD19^cre^ and CD19^cre^Foxo1^f/f^ mice by FACS. MZP B cells from CD19^cre^Foxo1^f/f^ mice were infected for 3 days with control lentivirus or Foxo1-IRES-EGFP-expressing lentivirus. ADAM28 fluorescence intensity was analyzed by FACS and ADAM28 mean fluorescence intensity (MFI) was shown. Data were analyzed by two tailed Student’s t test (**A**) Two-Way ANOVA plus Bonferroni post-tests (**B**) and One-Way ANOVA plus Dunnett’s Multiple Comparison Test (**C**), and shown as mean ± s.e.m. (N=12 for all groups) **P < 0.01.

**Fig. S7** Lack of Foxo1 promotes MZ B cell production from CD19-deficient MZP B cells. Both Foxo1 shRNA- and EGFP-expressing lentivirus-infected CD19-deficient MZP B cells (5 × 10^6^ cells/mouse) were i.v. injected into 7-week-old CD19-deficient mice (6 mice per group). Spleens were taken from recipient animals on days 7 after injection. (**A**) The percentage of IgM^hi^EGFP^+^ and CD21^hi^EGFP^+^ cells in total splenic lymphocytes, CD21 and CD23 expression between EGFP^+^ and EGFP^-^ B cells (**B**) and the percentage of IgM^hi^IgD^hi^, IgM^hi^IgD^lo^, CD1d^hi^CD23^+^, and CD1d^hi^CD23^-^ cells in EGFP^+^ B cells (**C**) were analyzed by FACS. (**A-C**) Data represent three independent experiments with three individual mice each.
